# Connectivity mapping uncovers small molecules that modulate neurodegeneration in Huntington’s disease models

**DOI:** 10.1007/s00109-015-1344-5

**Published:** 2015-10-02

**Authors:** Joshua L. Smalley, Carlo Breda, Robert P. Mason, Gurdeep Kooner, Ruth Luthi-Carter, Timothy W. Gant, Flaviano Giorgini

**Affiliations:** Department of Genetics, University of Leicester, Leicester, LE1 7RH UK; MRC Toxicology Unit, University of Leicester, Leicester, LE1 7HB UK; Department of Cell Physiology and Pharmacology, University of Leicester, Leicester, LE1 7RH UK; Centre for Radiation, Chemical and Environmental Hazards, Public Health England, Harwell Campus, Oxfordshire, OX11 0RQ UK

**Keywords:** Huntington’s, Huntingtin, Connectivity Map, cMap, Chemical screening, Therapeutic detection

## Abstract

**Abstract:**

Huntington’s disease (HD) is a genetic disease caused by a CAG trinucleotide repeat expansion encoding a polyglutamine tract in the huntingtin (HTT) protein, ultimately leading to neuronal loss and consequent cognitive decline and death. As no treatments for HD currently exist, several chemical screens have been performed using cell-based models of mutant HTT toxicity. These screens measured single disease-related endpoints, such as cell death, but had low ‘hit rates’ and limited dimensionality for therapeutic detection. Here, we have employed gene expression microarray analysis of HD samples—a snapshot of the expression of 25,000 genes—to define a gene expression signature for HD from publically available data. We used this information to mine a database for chemicals positively and negatively correlated to the HD gene expression signature using the Connectivity Map, a tool for comparing large sets of gene expression patterns. Chemicals with negatively correlated expression profiles were highly enriched for protective characteristics against mutant HTT fragment toxicity in in vitro and in vivo models. This study demonstrates the potential of using gene expression to mine chemical activity, guide chemical screening, and detect potential novel therapeutic compounds.

**Key messages:**

- Single-endpoint chemical screens have low therapeutic discovery hit-rates.
- In the context of HD, we guided a chemical screen using gene expression data.
- The resulting chemicals were highly enriched for suppressors of mutant HTT fragment toxicity.
- This study provides a proof of concept for wider usage in all chemical screening.

**Electronic supplementary material:**

The online version of this article (doi:10.1007/s00109-015-1344-5) contains supplementary material, which is available to authorized users.

## Introduction

Huntington’s disease (HD) is a neurodegenerative disease caused by a polyglutamine (polyQ) expansion in the huntingtin (HTT) protein [1]. HD is characterized by mood and cognitive impairments, motor dysfunction (including chorea) and, ultimately, death [2]. The molecular mechanisms underlying the cellular toxicity of mutant HTT are not entirely clear. However, both the loss of normal HTT function and the toxic gain of function of the mutant HTT protein are thought to be important in HD progression [3, 4]. A number of pathological mechanisms have been implicated in the onset and progression of HD, including mitochondrial dysfunction [5], neurotrophic factor deprivation [6], and excitotoxicity [7].

One of the most studied characteristics of mutant HTT is its propensity to form stable inclusions or aggregates [8]. These inclusions have been implicated in activating cellular stress mechanisms such as ER stress [9] and may play a role in protecting cells from mutant HTT-induced cell death [10]. Current evidence suggests that soluble mutant HTT oligomers produced early in the aggregation process are the major cause of HD neuropathology [11], and these have been shown to affect mitochondrial function [12]. However, mutant HTT aggregates can sequester a number of transcription factors which contain polyQ-rich regions—such as CBP [13], TBP [14] and SP1 [15]—both in the nucleus and in the cytoplasm. Sequestration of these proteins reduces the pool of transcription factors available to activate/repress their target genes [16], manifesting in transcriptional dysregulation that has been extensively characterized in human samples [17], in vivo [18, 19] and in vitro models [20, 21].

No drugs are currently available for delaying disease onset or progression in HD. In order to identify chemicals that might interfere with the molecular mechanisms of mutant HTT toxicity and prevent neurodegeneration, various high-throughput chemical screens have been performed in yeast [22], mammalian cells [23, 24] and cell-free assays [25]. In general, these screens have tested chemical libraries containing hundreds to thousands of chemicals for their effects on either aggregation of amino-terminal fragments of mutant HTT—which contain the polyQ stretch—or cellular apoptosis resulting from expression of these mutant HTT fragments. While such screens have identified benzothiazoles [25] and mTOR inhibitors [22] as potential therapeutic agents, these approaches have not yet been successfully translated to human therapies. One underlying issue may be the oversimplified nature of the screens, which focus on single endpoints.

We sought to identify chemicals that ameliorate mutant HTT fragment toxicity using gene expression signature-based chemical screening. Such approaches use a transcription pattern to describe a biological condition. By finding similarities and differences between the transcription patterns of various biological conditions, one can develop hypotheses regarding the similarities and differences of the biological conditions themselves. This approach has been used extensively in the cancer field to distinguish between cancer subtypes [26]. Linking chemical activity with gene expression has been demonstrated in yeast [27, 28], where compendiums of gene expression data for around 100 chemicals were created and hierarchical clustering used to group similar chemicals together with some success. More recently, chemical treatments in human cell lines were used to create a database for the Connectivity Map (cMap) [29]. The cMap is a technique which employs a transcriptional profile of interest to mine a database of gene expression data from 1600 chemical treatments and returns a measure of positive or negative similarity to each chemical as an output. By identifying similarities between the transcription patterns produced by different chemicals, functional similarities can be identified between those compounds. The major advantage of this approach is that this requires no prior understanding of the underlying biological processes at play, and is therefore unbiased. The initial aim of the cMap was to connect chemicals and diseases via similar transcriptional patterns. However, there are as yet few examples where a transcriptional pattern for a disease has been used as a ‘target’ for predicting potential therapeutic agents. The most notable employed a transcription pattern from fasting muscle tissue to identify urosolic acid as an inhibitor of skeletal muscle atrophy [30].

Transcriptional dysregulation in HD is well documented, making this disorder a particularly good paradigm for testing the disease relevance of the cMap technique. Using gene expression profiling data from human HD brains [17], we used the cMap to identify chemicals that induced positive (similar) or negative (inverse) transcriptional patterns to that of HD in an unbiased manner. The predicted chemicals were subsequently screened for neuroprotection in well-established acute mammalian cell and *Drosophila* models of HD that have previously been employed to detect chemicals that protect against mutant HTT toxicity [23, 31]. These HTT fragment-based models are well suited to chemical screening due to the rapid manifestation of a broad range of disease-relevant phenotypes with mechanistic relevance to HD [32], though they may not capture some disease phenotypes dependent upon full-length HTT protein. Using this approach, we identified an enriched list of compounds that ameliorate disease phenotypes in mammalian cell and *Drosophila* models of mutant HTT fragment toxicity, all of which produce an opposite transcription pattern to that observed in the HD patient samples. Positively correlated chemicals had no effect and served as an integrated control. These data provide a proof of concept and highlight the promise of the cMap for uncovering novel therapeutic strategies.

## Methods

### Tissue culture

PC12 cells stably transfected with a ponasterone A-inducible mutant HTT fragment (103Q) (first 17 amino acids of HTT plus polyQ repeat fused with green fluorescent protein (GFP); referred to as HTT103Q in the text)—known as the Htt14A2.5 cell line (Apostol et al. 2003)—were maintained and passaged in DMEM supplemented with 10 % horse serum (*v*/*v*), 5 % FCS (*v*/*v*) with 2 mM GlutaMax and 1 mM sodium pyruvate in T75 flasks (Greiner, UK) and incubated at 37 °C in a 5 % CO_2_ atmosphere.

### Drosophila husbandry, compound feeding and assays

Flies were maintained in standard maize food at 25 °C in light/dark cycle of 12:12. The *elavGAL4* [c155] fly stocks was obtained from the Bloomington Stock Center (Bloomington, IN). The HTT93Q line was a gift from Larry Marsh and Leslie Thompson [57]. Crosses were set up between male flies carrying *elavGAL4* driver and virgin females carrying the HTT93Q transgene. In the F1 generation only females expressed the HTT93Q, while males were used as controls.

Fly treatment, cell viability, caspase induction, aggregate counting and statistical analyses are described in the Supplementary Material.

## Results

### Connectivity mapping identifies chemicals predicted to modulate HD phenotypes

**Fig. 1:**
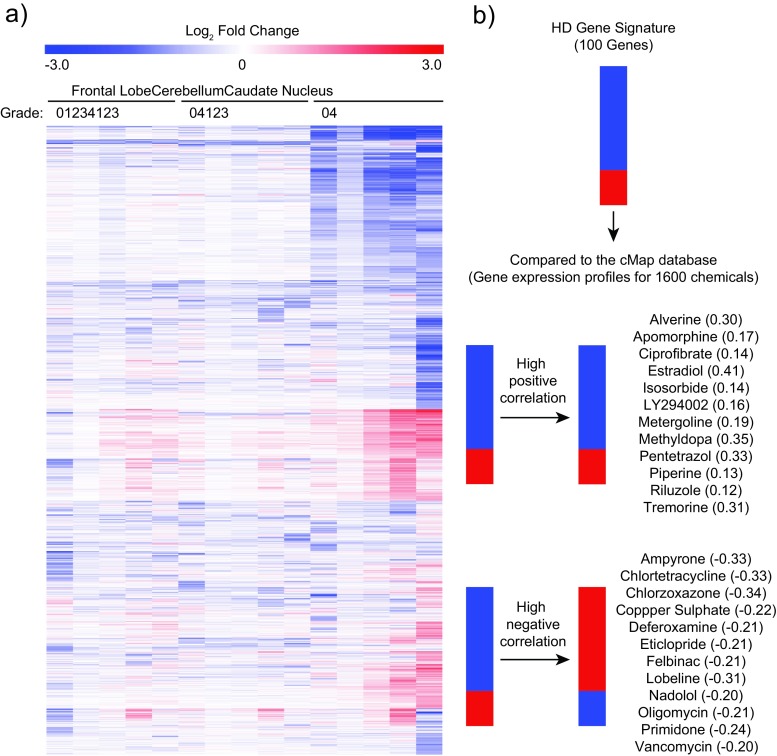
Using the Connectivity Map to identify chemicals with positive and negative connections to HD. **a** Vonsattel graded caudate nucleus, cerebellum, and frontal lobe from patients with HD were compared with age and sex-matched controls. HD-induced transcriptional dysregulation is most profound in the caudate nucleus, and transcriptional dysregulation increases with increasing Vonsattel grade. Genes not deemed significantly changed in any control/HD comparison were removed. The resulting gene matrix was hierarchically clustered using a Euclidian distance algorithm. **b** A gene signature for HD produces strong positive and negative connections with several chemicals. The top 100 most changed genes in the caudate nucleus grade 2 samples, selected by absolute fold change, were used to query the cMap gene expression database (Build 2). The top 12 most highly positively and negatively correlated chemicals were selected for further testing, where +1 and −1 represent a perfect positive or negative match, respectively

HD neuropathology has been classified using a grading system devised by Vonsattel et al. [33], which defines five grades of pathology (0–4) in increasing order of severity. We obtained gene expression data for several brain regions (frontal lobe, cerebellum, caudate nucleus) from HD patients at all Vonsattel grades, as well as age- and sex-matched control samples [17] from ArrayExpress (www.ebi.ac.uk/arrayexpress). HD samples were compared with age- and sex-matched controls, and significant gene expression changes were used to generate a heatmap to visually compare the regional and pathological alterations (Fig. 1a). This approach found the greatest amount of transcriptional dysregulation occurs in the caudate nucleus compared to other regions. We found that while the genes dysregulated across grades 0–2 are very similar, the degree of dysregulation increases with increasing Vonsattel grade, as originally described [17]. In order to use samples with a robust gene expression changes, we chose data from the caudate nucleus at grade 2 to generate a gene signature for HD. By using grade 2 samples, we avoided gene expression changes linked with the gross pathological events found at later stages of the disease (grades 3 and 4). We selected the most dysregulated differentially expressed genes (DEGs) by absolute fold change, creating a 100 gene signature to query the cMap for chemicals that produced positive or negative gene expression patterns (Table S1). The highest scoring positive and negative connections were identified (a linear scoring system where +1 and −1 represent a perfect positive and negative correlation, respectively), with 12 in each category selected for downstream testing (Fig. 1b). Notably, these candidate chemicals represented a diverse set of pharmacological classes, including endogenous hormones (estradiol), PI3K-inhibitors (LY294002), β-blockers (nadolol), metal chelators (deferoxamine) and metabolic inhibitors (oligomycin).

### Inversely connected chemicals reduce mutant HTT-induced caspase activation

**Fig. 2:**
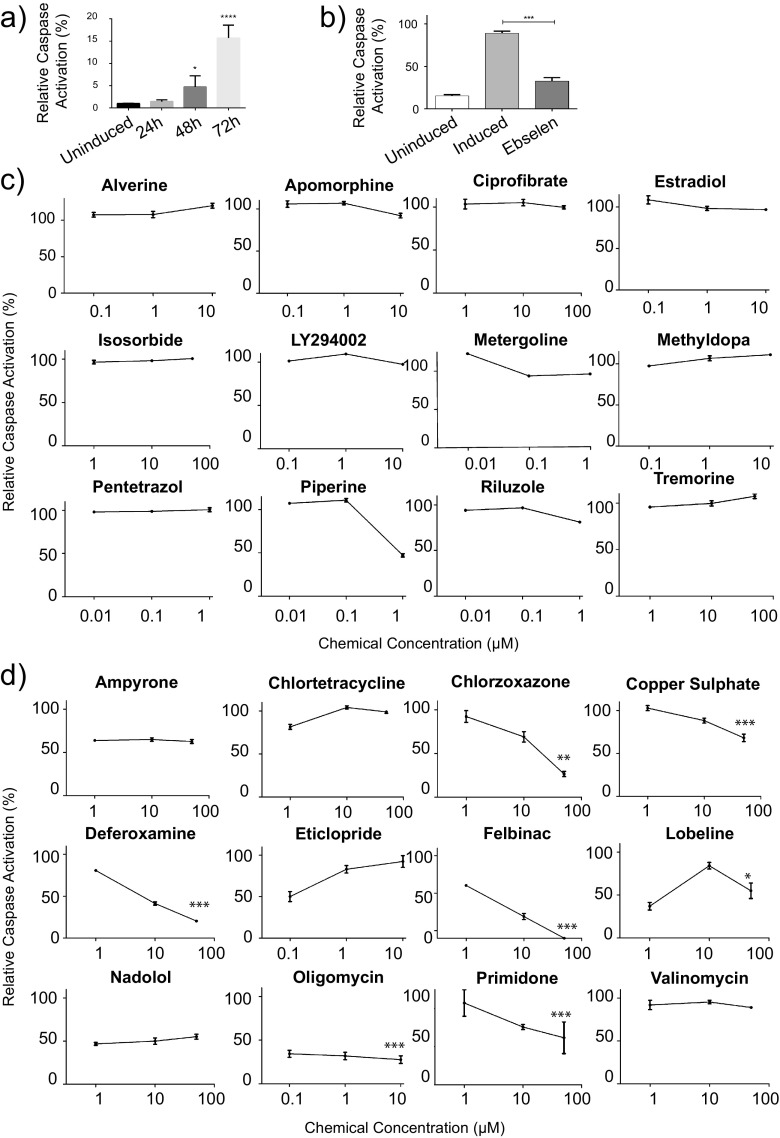
Negatively correlated chemicals ameliorate mutant HTT fragment toxicity in PC12 cells. **a** Caspase activity measurements for chemical screening were carried out at 72 h post-HTT103Q-induction as caspase activity peaks at this time point. **b** All test plates contained a 10 μM ebselen treatment. Ebselen is known to prevent caspase 3/7 cleavage post HTT103Q induction, and therefore served as a positive control [31]. **c** Chemicals that induce gene expression changes that positively correlate with HD have no significant effect on caspase 3/7 activation in PC12 cells expressing HTT103Q. **d** Seven of the 12 selected chemicals that induce gene expression changes which were negatively correlated with HD reduce HTT103Q toxicity in PC12 cells. PC12 cells expressing HTT103Q were exposed to sub-cytotoxic concentrations of chemicals for 72 h. Mean ± SEM (*N* = 3). *P < 0.05, **P < 0.01, ***P < 0.001

We next used a luminescent caspase-activation assay to assess whether any of the chemicals with positively or negatively correlated signatures modulated mutant HTT-induced apoptosis in a PC12 cell line which inducibly expresses an N-terminal mutant HTT fragment (HTT103Q), which we and others have previously employed [20, 31]. A range of sub-cytotoxic concentrations, determined by MTS cellular toxicity assays (Fig. S1), were added simultaneously with the induction of HTT103Q expression, and caspase 3/7 activation was measured after 72 h, a point at which substantial caspase 3/7 induction is observed post-induction (Fig. 2b). The antioxidant ebselen was included on every plate as a positive control [31] (Fig. 2a). Chemicals with positively correlated expression signatures had very little effect on caspase activation over the 72-h incubation period (Fig. 2c). Piperine produced a reduction in caspase activation; however, this was due to the highest concentration being close to the cytotoxic window (Fig. S1). In contrast, 7 of the 12 chemicals with negatively correlated signatures significantly reduced HTT103Q-induced caspase activation (Fig. 2d). Chlorzoxazone, copper sulphate, deferoxamine, felbinac, oligomycin and primidone all reduced caspase activation in a dose-dependent manner. These chemicals were subsequently selected for further investigation.

### Deferoxamine and oligomycin reduce mutant HTT inclusion body formation

**Fig. 3:**
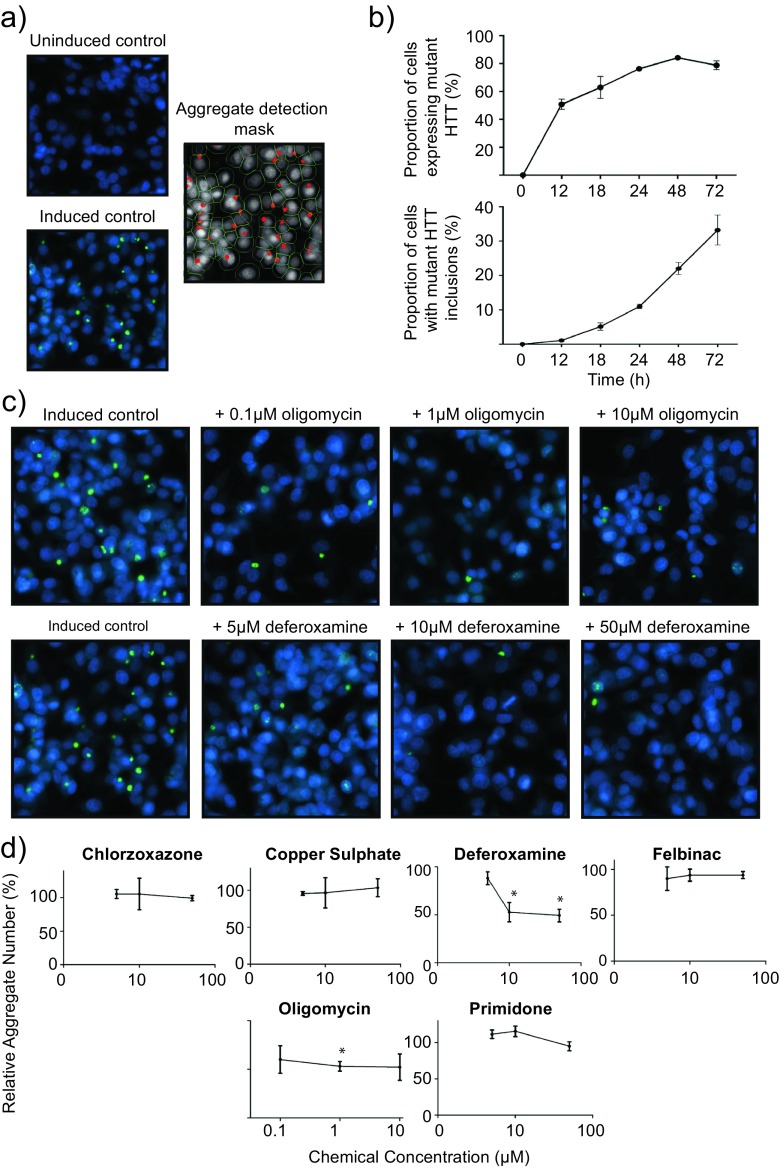
Protective chemicals differentially modulate HTT103Q aggregation. **a** High-throughput recording of HTT103Q aggregation in PC12 cells using Cellomics imaging technology. PC12 cells expressing GFP-tagged HTT103Q were stained with Hoechst stain to visualize the nucleus. A Cellomics ‘spotcount’ algorithm was used to identify cell nuclei, estimate cell boundaries, detect intracellular HTT103Q aggregates and record their intensity. **b** PC12 cells express HTT103Q extensively following induction with ponasterone A, detected by GFP signal, and stable HTT103Q aggregates form in the cells. A time point of 48 h was used for further experiments as it allowed for either an increase or decrease in aggregation following chemical treatment. **c** Representative images of HTT103Q aggregates following induction and exposure to deferoxamine or oligomycin. **d** Deferoxamine and oligomycin reduce HTT103Q aggregation in PC12 cells. PC12 cells expressing GFP-tagged HTT103Q were exposed to chemicals that reduced caspase 3/7 activation for 48 h. The cells were fixed, Hoechst stained, and HTT aggregates counted using Cellomics automated counting algorithms. Mean ± SEM (*N* = 3). *P < 0.05

Mutant HTT aggregation is an important early, upstream process in the development of HD. To gain mechanistic insight into whether the protective chemicals modulated HTT103Q inclusion body formation, we employed a robust fluorescence-based mutant HTT aggregation assay in living cells. A Cellomics Arrayscan High Content Screening system with an aggregate counting algorithm was used to quantify GFP-tagged HTT103Q aggregates in the PC12 cell model of mutant HTT fragment toxicity described above (Fig. 3a). Predefined concentrations of the chemicals which displayed significant dose–response relationships in the caspase 3/7 assay were added simultaneously with HTT103Q induction, and the number of aggregates was recorded 48 hours after the induction of mutant HTT fragment expression, the point at which maximal HTT expression occurs based upon GFP signal and a steady-state rate of aggregate formation is observed (Fig. 3b).

Of the seven compounds tested, only deferoxamine and oligomycin significantly altered the formation of HTT103Q-containing inclusion bodies (Fig. 3c, d). Deferoxamine treatment yielded a dose-dependent reduction in the number and the intensity of GFP-tagged HTT103Q aggregates in PC12 cells. While no effect was observed at 5 μM, a significant reduction in aggregation was observed at 10 and 50 μM (*P* < 0.05 and *P* < 0.01, respectively), with a maximal reduction of ~50 % (Fig. 3d). Oligomycin also reduced the number of HTT103Q aggregates by ~50 %, with the most robust effect observed at the concentration of 1 μM (*P* < 0.05) (Fig. 3d). These data highlight that the chemicals identified by the cMap likely have varied mechanisms of protection, as all of the chemicals tested for effects on HTT103Q aggregation prevented HTT103Q-induced toxicity, yet only oligomycin and deferoxamine had a notable effect on HTT103Q aggregation.

### Chlorzoxazone and deferoxamine ameliorate neurodegeneration in mutant HTT expressing fruit flies

**Fig. 4:**
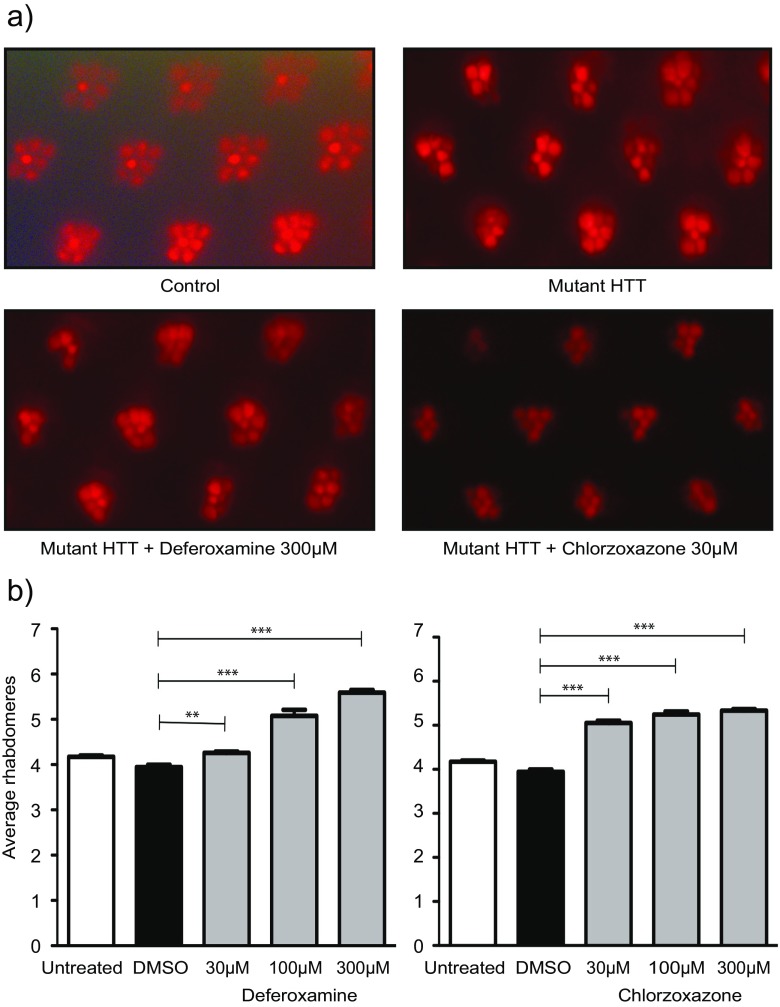
Deferoxamine and chlorzoxazone ameliorate neurodegeneration in flies expressing a mutant HTT fragment. *Drosophila* expressing HTT93Q exon 1 pan-neuronally were exposed to either deferoxamine or chlorzoxazone in their food at the concentrations shown for 7 days. Rhabdomeres were scored via the pseudopupil assay. **a** Representative images of control and treated *Drosophila* rhabdomeres. **b** Both deferoxamine and chlorzoxazone significantly increased rhabdomere number in HTT93Q exon 1-expressing flies. Data represent the mean rhabdomere count per ommatidium ± SEM (*N* = 12). **P < 0.01, ***P < 0.001

We next sought to validate the cMap approach in vivo using fruit flies expressing a mutant HTT fragment. We selected chlorzoxazone and deferoxamine for further testing as they were both highly protective in PC12 cells but differentially affected mutant HTT aggregation. In addition, chlorzoxazone and deferoxamine are currently used therapeutically in humans as a skeletal muscle relaxant [34] and iron chelator [35], respectively. We employed a widely studied fruit fly model of mutant HTT toxicity, in which the human exon 1-encoded fragment of HTT (HTT93Q exon 1) is expressed pan-neuronally (via the *elavGAL4* driver) using the bitransgenic GAL4/UAS system [36]. These flies exhibit several phenotypes that recapitulate HD symptoms, including neurodegeneration, locomotor impairments, and reduced lifespan [31, 37]. Neurodegeneration can be robustly assessed by scoring the number of photoreceptor neurons—known as rhabdomeres—present in the fly eye. The compound eyes of *Drosophila* contain repeating units of ommatidia, each of which contain eight rhabdomeres, seven of which are visible via the pseudopupil assay. Newly emerged fruit flies were fed with food containing 30, 100 or 300 μM of chlorzoxazone or deferoxamine for 7 days. Strikingly, both compounds significantly reduced rhabdomere degeneration in HTT93Q exon 1 flies compared to the controls (Fig. 4a, b). Deferoxamine ameliorated degeneration in a dose-dependent manner, with a modest yet significant reduction in photoreceptor loss at 30 μM (*P* < 0.01), which became more pronounced at 100 and 300 μM (*P* < 0.001) (Fig. 4b). Chlorzoxazone significantly reduced neurodegeneration in HTT93Q exon 1 flies at all concentrations tested (*P* < 0.001) (Fig. 4b). This work indicates that these compounds confer neuroprotection in HTT93Q exon 1 fruit flies, and provide in vivo support for our findings in mammalian cells.

### The modulation of HD gene expression divides negatively correlated chemicals into two distinct groups

**Fig. 5:**
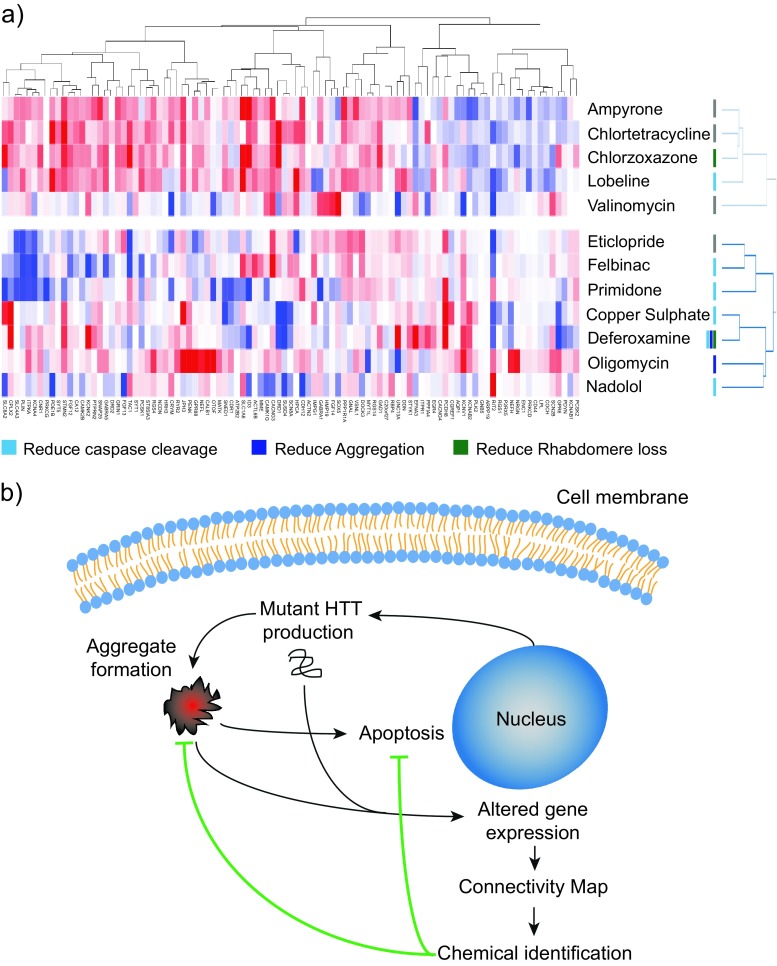
Scheme indicating that a gene expression pattern for HD can be used to identify chemicals that ameliorate mutant HTT fragment-mediated cell damage. **a** Hierachical clustering of the effect of each negatively correlated chemical on HD-induced genes reveals two distinct clusters of chemicals. Protective chemicals are contained within both clusters, with those effecting aggregation in the lower cluster. This highlights how the cMap can identify chemicals with multiple modes of action that protect against mutant HTT toxicity. The data was extracted from the cMap database and was clustered by Pearson correlation. **b** A human-derived gene expression profile for HD was used to identify potential therapeutic agents. Several chemicals reduced HTT103Q-induced cell death in vitro and in vivo, both with and without affecting aggregation, thus indicating the detection of protective chemicals with different mechanisms of action

We next extracted the gene expression data for each of the negatively correlated chemicals from the cMap database and used hierarchical clustering to investigate their effects on HD-induced genes. Hierachical clustering revealed two distinct groups of chemicals, one containing chlorzoxazone, which rescued rhabdomeres in HTT93Q exon 1 flies but had no effect on HTT103Q aggregation in PC12 cells (Fig. 5a). The other group contained deferoxamine and oligomycin, both of which reduced HTT103Q aggregation in PC12 cells, with deferoxamine also preventing rhabdomere loss in HTT93Q exon 1 flies. This illustrates that the use of the cMap technique in this manner can identify chemicals with similar characteristics but differing mechanisms of action. The heatmap also illustrates how each gene contributed to the identification of the chemical as being negatively correlated to HD in our original analysis above.

## Discussion

Our primary aim in this study was to employ the cMap approach to predict chemicals that may protect against mutant HTT-induced toxicity. In order to achieve this, we employed a comprehensive microarray dataset for HD and control human brain samples [17] to generate the HD “gene signature.” The largest numbers and magnitudes of transcriptional changes observed were present in the caudate nucleus [17] (Fig. 1a), corresponding with evidence indicating that the caudate nucleus is one of the earliest and most affected brain regions in HD [33, 38]. However, it should be noted that as many as 50 % of the caudate nucleus neurons may be lost by grade 2 [39]—which undoubtedly contributed to the transcriptional changes observed here. Nonetheless, the gene expression changes showing the most extreme magnitudes of down-regulation in HD—which have been validated by laser-capture microdissection—indicate these likely reflect alterations in expression at the mRNA/cell level [17].

The cMap database was queried with the gene signature derived from the human HD data. There is an obvious disconnect between the characteristics of neuronal cells in vivo and the cultured immortalized cells used to construct the cMap database. However, in both cases, the disease/chemical induced gene expression was compared with a precise control, therefore minimizing the contribution of cell-specific gene expression. Differing cell machinery would still influence the transcriptional response to a disease or chemical stimulus. Despite this, it was recently demonstrated using the cMap that a gene expression profile for skeletal muscle atrophy generated from mouse muscle tissue was able to identify ursolic acid as a compound whose transcriptomic effect was inversely correlated to that of disease, and which subsequently was found to reduce muscle atrophy in this mouse model [30]. This not only demonstrates the potential of the cMap to identify connections between diseases and chemicals across the in vivo/in vitro boundary, but also with differences in time course and species.

We hypothesized that the HD gene signature reflected a mixture of gene expression changes due to mutant HTT toxicity as well as protective cellular response(s). Therefore, both positively and negatively correlated chemicals were selected in an unbiased manner and screened for effects on mutant HTT fragment toxicity. Of the 12 positive chemical connections—whose expression signatures correlated with those of HD—none significantly reduced HTT103Q toxicity. In contrast, 7 of the 12 chemicals whose expression signatures negatively correlated with those of HD significantly reduced HTT103Q-induced caspase activation (Fig. 2). Here, the positively correlated group of chemicals also serves as a control to emphasize that the negatively correlated chemicals are truly enriched for protective characteristics. These data suggest that several of the corresponding genes in the HD signature reflect the toxicity of mutant HTT, corroborating the idea that significant transcriptional changes observed in the human HD gene signature are caused by the dysfunction of functionally important transcription factors [40, 41].

The role of HTT aggregates in HD pathogenesis is controversial. There is evidence that HTT aggregates cause cellular stress and cell death by apoptosis [9], as well as conferring a protective mechanism [10]. Indeed, various chemicals that either increase or decrease HTT aggregation have been shown to be cytoprotective [24]. Nonetheless, aggregation of mutant HTT is an important hallmark of HD pathology that provides insight into pathogenesis. With this in mind, we tested whether seven chemicals that reduced HTT103Q toxicity in PC12 cells could modulate HTT103Q aggregation, and found that both oligomycin and deferoxamine significantly reduced HTT103Q aggregation (Fig. 3). Oligomycin is an inhibitor of ATP synthase (complex V) that utilizes the mitochondrial membrane proton gradient to convert ADP to ATP [42]. Oligomycin, as well as other metabolic inhibitors, have previously been shown to rescue cell death in HD models [43]. It is thought that such inhibitors reduce cell death by activating the pro-survival ERK and AKT pathways [43]. Although the ability of oligomycin to rescue mutant HTT-induced cell death has previously been documented, its effect on mutant HTT aggregation has not. Unfortunately, oligomycin is extremely toxic in vivo in mammals, causing metabolic acidosis and nephrotoxicity [44]. Thus, while the identification of oligomycin via the cMap validates this approach, it would not represent a viable therapeutic compound.

Deferoxamine (or desferal) is a bacterial siderophore with a high affinity for iron and has antioxidant properties [45], which is used therapeutically as an iron-chelating agent. Short exposure to deferoxamine is an effective treatment for acute iron overdose [46] and is used as a longer-term treatment for patients with blood transfusion-related iron overload [47]. Deferoxmine has also been used to protect against both lipopolysaccharide- and doxorubicin-induced toxicity [48, 49], where reactive oxygen species (ROS) are known to play an important role. Oxidative stress has been implicated in mutant HTT-induced toxicity, with lipid peroxidation products such as malondialdehyde observed in HD-affected regions of the brain [50]. Here, we found that deferoxamine reduced mutant HTT toxicity and aggregation in the PC12 cell model, and as well as ameliorating neurodegeneration in mutant HTT-expressing flies. This complements previous work showing HTT inclusion bodies contain iron, and chelators such as deferoxamine alter aggregate dynamics [51] and are neuroprotective. This suggests that deferoxamine is effective upstream of HTT-aggregation but may also be protective due to its antioxidant properties, similar to our positive control, ebselen.

Chlorzoxazone significantly reduced HTT-induced caspase activation in PC12 cells and rhabdomere loss in flies expressing HTT93Q exon 1, while having no effect on HTT aggregation. Chlorzoxazone is a clinically used skeletal muscle relaxant which relieves discomfort due to muscle spasm [52]. Previous work has suggested that chlorzoxazone reduces excitatory neurotransmission by modulating calcium-activated SK potassium channels [53]. Interestingly, chlorzoxazone has been proposed to counteract the neurologic effects of CACNA1A mutations [54]. Chlorzoxazone also has anti-inflammatory properties [55], which may also be relevant for HD, where neuroinflammation has been heavily implicated [56].

In this study, we have thus validated the effectiveness of the cMap approach to establish therapeutic relationships between chemicals and HD based upon gene expression profiles. We have demonstrated that by using a gene signature for HD, the cMap can identify potential therapeutic agents with a hit rate that far surpasses typical phenotypic screens. Excitingly, we were able to identify protective chemicals with multiple modes of action, a feature that would have been missed in a phenotypic screen of mutant HTT fragment aggregation alone. It is also noteworthy that the multidimensionality of a whole genome gene expression assay allows the identification of chemicals for multiple characteristics—meaning the same data could be used for multiple disease screens. Thus, this work serves as a proof of concept for such screens to be extended to other diseases where transcriptional dysregulation is pivotal, and also for further HD-related screens to be carried out with larger gene-expression datasets (Fig. 5), for instance in mutant-HTT expressing cell lines. Indeed, a greatly expanded version of the cMap database is soon to be released to the scientific community which utilizes the L1000 gene expression measurement method (http://www.broadinstitute.org/LINCS/). The database will be dramatically enlarged from ~6000 profiles for 1300 chemicals in four cell lines, to ~576,000 profiles for 4000 chemicals, 9000 gene knockdowns and 3000 gene overexpressions in ten cell lines. Thus, it is clear that the power of the cMap to identify chemicals/genes that modulate disease phenotypes will be greatly increased. The work presented here also forms a strong rationale for the inclusion of a database with mutant HTT-expressing cell treatments in future cMap iterations. This new resource—combined with the interrogation of additional gene expression data sets at multiple pathological stages for HD and other diseases—will provide a powerful tool for identification of candidate therapeutic chemicals for these disorders.

## Electronic supplementary material

ESM 1 (pdf 524 kb)
