# Supplementary material for: Connectivity mapping uncovers small molecules that modulate neurodegeneration in Huntington’s disease models
Source: J Mol Med (Berl). 2015 Oct 2;94:235–45. doi: 10.1007/s00109-015-1344-5 (PMC4762922; doi:10.1007/s00109-015-1344-5)
Supplement: Supplementary file 1 — (pdf 524 kb) [file 109_2015_1344_MOESM1_ESM.pdf]

Electronic Supplementary Material (ESM)

J Mol Med 2015

Title:

Connectivity mapping uncovers small molecules that modulate neurodegeneration in Huntington's disease models

Authors:

Joshua L. Smalley[1,2], Carlo Breda[1], Robert P. Mason[1], Gurdeep Kooner[1], Ruth Luthi-Carter[3], Timothy W. Gant[2,4], Flaviano Giorgini[1*]

[1] Department of Genetics, University of Leicester, Leicester, LE1 7RH, UK

[2] MRC Toxicology Unit, University of Leicester, Leicester, LE1 7HB, UK

[3] Department of Cell Physiology and Pharmacology, University of Leicester, Leicester, LE1 7RH, UK

[4] Centre for Radiation, Chemical and Environmental Hazards, Public Health England, Harwell Campus, Oxfordshire OX11 0RQ, UK

Contact:

Prof. Flaviano Giorgini,

Department of Genetics, University of Leicester, Leicester, LE1 7RH, UK

Email: fg36@le.ac.uk

Telephone: +44 116 252 3485

Materials and Methods

Drosophila compound feeding and assays

For drug feeding experiments, maize media was heated until liquid and distributed into vials. Deferoxamine or chlorzoxazone (Sigma Aldrich, UK) were freshly prepared in DMSO as 1000 X stocks and added to the media. Newly emerged HTT93Q exon 1 flies were transferred to vials containing control or treated food, which was changed daily for 7 days. At day 7, flies were anaesthetized with CO_2, their heads removed and mounted face-up on microscope slides. A Nikon Optiphot-2 microscope at 40 X magnification was used for counting rhabdomeres from approximately 100 ommatidia per fly, and 12 flies per treatment.

Microarray analysis

Microarray data for diagnosed HD and control postmortem brain samples [1] was obtained from ArrayExpress (www.ebi.ac.uk/arrayexpress) and build 2 of the cMap database. CEL files were imported into ArrayTrack software [2] and summarized using aspects of the MAS5 algorithm, where the background fluorescence is subtracted and each mismatch probe intensity is subtracted from its respective perfect match probe [3]. The resulting expression data was normalized using a quantile scaling method [4] and samples compared using a Welch t-test. The entire dataset was filtered to remove genes statistically unchanged any genes with a mean channel intensity (MCI) lower than 50, as a feature of the MAS5 summarization is high false positives at low MCIs due to the subtraction of sometimes high mismatch probe intensities [3]. Visualization and hierarchical clustering of the dataset was carried out by Euclidian distance (Figure 1) or Pearson correlation (Figure 5) using GENE-E software (www.broadinstitute.org). A gene expression signature for HD was defined by selecting the most changed genes from grade 2 caudate nucleus of HD versus

control. The top 100 most differentially expressed genes were selected by absolute log_2 fold change once the dataset was filtered for significance ($P < 0.05$). This method of gene selection was chosen in order to mimic best the manner in which genes are ordered in cMap reference database. The gene signature was used to query build 2 of the cMap dataset [5]. The algorithm used to carry out the query was that produced by Zhang and Gant [6,7]. Equal weighting is applied to each gene in the gene signature, which is compared to around 3000 profiles in the cMap database, within which each gene is ranked in a linear manner according to absolute fold change, where an up-regulation is positive and a down-regulation is negative. The similarity score is determined by adding the scores for each gene in the gene signature and dividing it by the maximum possible score. Perfect positive and negative scores are therefore 1 and -1 respectively.

Cell viability

Cell viability was measured using an MTS assay kit (Promega, UK) according to the manufacturer's instructions. Cells were seeded in a 96-well plate (Greiner, UK) at a density of 5 x 10^3 cells per well in 100 µl of medium. The cells were allowed to adhere for 24 hours prior to treatment with several concentrations of each chemical dissolved in DMSO (final concentration 0.1 %) for 72 hours. Following treatment, the media was aspirated, and pre-warmed fresh media added to each well. 20 µl of MTS assay reagent was added to each well and incubated at 37 °C and 5 % CO_2 for 3 hours before the absorbance at 490 nm was measured using a Wallac Victor^2 spectrophotometer. Values were normalized to the DMSO control.

Caspase 3/7 assay

Caspase activation was determined using a Caspase-Glo 3/7 assay kit (Promega, UK) according to the manufacturer's instructions. PC12 cells were seeded in a white 96-well plate (Greiner, UK) at a density of 5 x 10^3 cells per well in 100 µl of medium. The cells were allowed to adhere for 24 hours prior to treatment. Induced and uninduced cells were treated with a range of sub-cytotoxic chemical concentrations. Induced cells were concomitantly treated with 5 µM ponasterone A for 72 hours. Following treatment, 50 µl of Caspase-Glo reagent was added to each well. The plate was shaken for 30 seconds at 300 rpm and incubated in the dark at room temperature for 1 hour. The luminescence of each sample was measure using a BMG Labtech FLUOstar Omega luminometer / spectrophotometer. Assays were performed in triplicate and values normalized to uninduced (minimum) and induced (maximum) controls. Ebselen, an antioxidant with documented protective effects [8] was included as a positive control on every plate.

Cellomics assay

PC12 cells were seeded in a 24-well plate at 1 x 10^4 cells per well and allowed to adhere for 24 hours prior to treatment. The cells were treated with a range of sub-cytotoxic chemical concentrations, and induced with ponasterone A for 48 hours. The cell media was removed and the cells fixed using 4 % paraformaldehyde in PBS for 10 minutes at room temperature. The paraformaldehyde was removed and the cells stained with a 50 µg/ml solution of Hoechst 33342 in BSA for 10 minutes at room temperature. The Hoechst solution was removed, 1 ml of PBS added, and samples stored at 4°C until analysis. The plate was transferred to the Cellomics machine and the 'Spotcount' bioapplication used to detect HTT aggregates. Hoechst-stained nuclei were detected in channel 1 with a 386 nm filter, and a mask fitted to define the area of

the nucleus and predict the cell boundary. GFP labeled aggregates above an intensity threshold within the cytoplasmic area were counted in channel 2 with a 485 nm filter. 4000 cells/well were counted in randomly selected fields containing >50 cells, with a maximum of 100 fields per well. Values were normalized to the induced control.

Statistical Analyses

For all non-microarray-based experiments, statistical significance was measured using a one-way ANOVA and the results were a product of 3 biological replicates (N = 3) unless otherwise stated. The P value threshold for statistical significance for all experiments was 0.05.

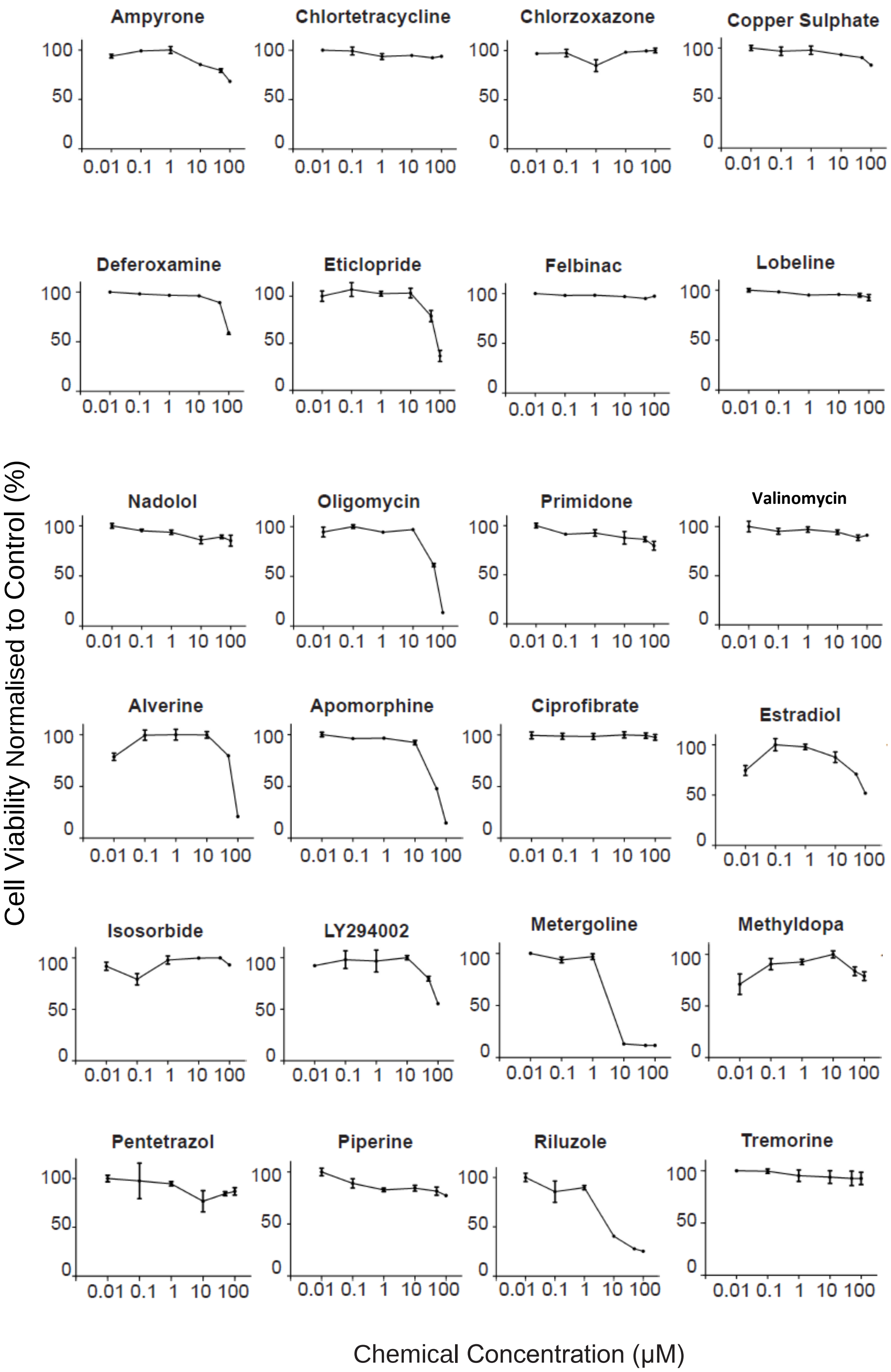

Figure S1. Chemicals that induced transcriptional changes which correlated or were inverse to the HD gene signature were tested for cytotoxicity in PC12 cells. The cells were exposed to the chemicals shown for 72 hours (10 nM - 100 µM). Cell viability was determined by MTS assay. The data represents mean ± SEM (N = 3).

Table S1. The 100 largest gene-expression changes in the caudate nucleus of Grade 2 HD patients compared to age and sex-matched controls. These data were used to create a gene signature to query the Connectivity Map. Microarray data was obtained from a publically available dataset (E-GEOD-3790). Data was normalized by mean scaling and filtered for genes with a mean fluorescent channel intensity greater than 50 and deemed significantly changed ($P < 0.05$). The top 100 most changed genes were selected by highest absolute fold change.

**Gene Name**	**Description**	**REFSEQ**	**Fold Change**
SNED1	sushi, nidogen and egf-like domains 1	NM_001080437	5.34
CD44	cd44 molecule (indian blood group)	NM_000610	5.13
PLIN	perilipin	NM_001145311	4.60
ID3	inhibitor of dna binding 3, dominant negative helix-loop-helix protein	NM_002167	4.56
AQP1	aquaporin 1	NM_001185060.1	4.38
SOX5	sry (sex determining region y)-box 5	NM_006940	3.97
RGS1	regulator of g-protein signaling 1	NM_002922	3.92
HIST1H3D	histone cluster 1, h3d	NM_003531	3.84
RGS4	regulator of g-protein signaling 4	NM_001102445	-20.75
CACNG3	calcium channel, voltage-dependent, gamma subunit 3	NM_006539	-12.53
RGS14	regulator of g-protein signaling 14	NM_006480	-10.56
KCNV1	potassium channel, subfamily v, member 1	NM_014379	-9.89
MME	membrane metallo-endopeptidase	NM_000902	-9.78
ACTN2	actinin, alpha 2	NM_001103	-9.51
KCNAB2	potassium voltage-gated channel, shaker-related subfamily, beta member 2	NM_001199860	-9.13

KCNAB1	potassium voltage-gated channel, shaker-related subfamily, beta member 1	NM_003471	-7.89
CAMK1G	calcium/calmodulin-dependent protein kinase ig	NM_020439	-7.64
GRIN1	glutamate receptor, ionotropic, n-methyl d-aspartate 1	NM_000832	-7.50
PRKCG	protein kinase c, gamma	NM_002739	-7.35
SLC4A3	solute carrier family 4, anion exchanger, member 3	NM_005070	-6.96
PDE1B	phosphodiesterase 1b, calmodulin-dependent	NM_000924	-6.75
NCDN	neurochondrin	NM_001014839	-6.61
ACTL6B	actin-like 6b	NM_016188.4	-6.57
IMPG1	interphotoreceptor matrix proteoglycan 1	NM_001563	-6.46
GNAL	guanine nucleotide binding protein, alpha activating activity polypeptide, olfactory type	NM_001142339	-6.27
STYK1	serine/threonine/tyrosine kinase 1	NM_018423	-6.27
DDN	dendrin	NM_015086	-6.11
CALB1	calbindin 1, 28kda	NM_004929	-5.93
HRH3	histamine receptor h3	NM_007232	-5.87
NEFL	neurofilament, light polypeptide	NM_006158	-5.80
GAD1	glutamate decarboxylase 1 (brain, 67kda)	NM_000817	-5.73
NEFH	neurofilament, heavy polypeptide	NM_021076	-5.61
CACNG4	calcium channel, voltage-dependent, beta 4 subunit	NM_000726	-5.57
HPCA	hippocalcin	NM_002143	-5.43
FGF14	fibroblast growth factor 14	NM_004115	-5.32
GPR6	g protein-coupled receptor 6	NM_005284	-5.28
EGR4	early growth response 4	NM_001965	-5.26
P2RX5	purinergic receptor p2x, ligand-gated ion channel, 5	NM_001204519	-5.18
DRD2	dopamine receptor d2	NM_000795	-5.17
EFNA3	ephrin-a3	NM_004952	-5.17
FGF13	fibroblast growth factor 13	NM_001139498	-5.14

PDYN	prodynorphin	NM_001190892	-5.10
GABRA1	gamma-aminobutyric acid (gaba) a receptor, alpha 1	NM_000806	-5.09
GABRA5	gamma-aminobutyric acid (gaba) a receptor, alpha 5	NM_000810	-5.04
OTOF	otoferlin	NM_004802	-5.03
PLK2	polo-like kinase 2	NM_006622	-5.03
RIT2	ras-like without caax 2	NM_002930	-5.03
COCH	coagulation factor c homolog, cochlin (limulus polyphemus)	NM_001135058	-5.00
FGF12	fibroblast growth factor 12	NM_004113	-4.97
ATP2B2	atpase, ca++ transporting, plasma membrane 2	NM_001001331	-4.93
CA12	carbonic anhydrase xii	NM_001218	-4.93
SCN2B	sodium channel, voltage-gated, type ii, beta	NM_004588	-4.92
TAC1	tachykinin, precursor 1	NM_003182	-4.91
SLC8A2	solute carrier family 8 (sodium/calcium exchanger), member 2	NM_015063	-4.89
KCNK2	potassium channel, subfamily k, member 2	NM_001017424	-4.83
ARPP-19	camp-regulated phosphoprotein, 19kda	NM_006628.4	-4.83
C20orf27	chromosome 20 open reading frame 27	NM_001039140	-4.79
RBP4	retinol binding protein 4, plasma	NM_006744	-4.78
PCSK1	proprotein convertase subtilisin/kexin type 1	NM_000439	-4.76
KCNA4	potassium voltage-gated channel, shaker-related subfamily, member 4	NM_002233	-4.64
ITPKA	inositol-trisphosphate 3-kinase a	NM_002220	-4.53
SNAP25	synaptosomal-associated protein, 25kda	NM_003081	-4.52
ITPR1	inositol 1,4,5-trisphosphate receptor, type 1	NM_001099952	-4.51
HMP19	hmp19 protein	NM_015980	-4.49
UNC13A	unc-13 homolog a (c. elegans)	NM_001080421	-4.39
SYT1	synaptotagmin i	NM_001135805	-4.36
VSNL1	visinin-like 1	NM_003385	-4.36
PCSK2	proprotein convertase subtilisin/kexin type 2	NM_001201528	-4.30

DOCK3	dedicator of cytokinesis 3	NM_004947	-4.27
NRGN	neurogranin (protein kinase c substrate, rc3)	NM_001126181	-4.22
A2BP1	rna binding protein, fox-1 homolog (c. elegans) 1	NM_145893.2	-4.21
ENC1	ectodermal-neural cortex 1 (with btb-like domain)	NM_003633	-4.21
GPR88	g protein-coupled receptor 88	NM_022049	-4.19
RYR2	ryanodine receptor 2 (cardiac)	NM_001035	-4.19
DRD1	dopamine receptor d1	NM_000794	-4.18
SCN8A	sodium channel, voltage gated, type viii, alpha subunit	NM_001177984	-4.14
SLC1A6	solute carrier family 1 (high affinity aspartate/glutamate transporter), member 6	NM_005071	-4.13
PTPRN2	protein tyrosine phosphatase, receptor type, n polypeptide 2	NM_002847	-4.13
SYT5	synaptotagmin v	NM_003180	-4.12
STMN2	stathmin-like 2	NM_001199214	-4.10
PPP1R1A	protein phosphatase 1, regulatory (inhibitor) subunit 1a	NM_006741	-4.09
CGREF1	cell growth regulator with ef-hand domain 1	NM_001166239	-4.05
CNR1	cannabinoid receptor 1 (brain)	NM_001160226	-4.04
CAMK2B	calcium/calmodulin-dependent protein kinase ii beta	NM_001220	-3.98
CDH12	cadherin 12, type 2 (n-cadherin 2)	NM_004061	-3.96
CRYM	crystallin, mu	NM_001014444	-3.95
PPFIA4	protein tyrosine phosphatase, receptor type, f polypeptide, interacting protein, alpha 4	NM_015053	-3.94
GNB5	guanine nucleotide binding protein (g protein), beta 5	NM_006578	-3.94
LRRC37B2	leucine rich repeat containing 37b pseudogene 2	NR_015341	-3.94
PRKCD	protein kinase c, delta	NM_006254	-3.93
ST8SIA3	st8 alpha-n-acetyl-neuraminide alpha-2,8-sialyltransferase 3	NM_015879	-3.93
CDR1	cerebellar degeneration-related protein 1, 34kda	NM_004065	-3.92
SUSD4	sushi domain containing 4	NM_001037175	-3.91
PENK	proenkephalin	NM_001135690	-3.90

MYT1L	myelin transcription factor 1-like	NM_015025	-3.87
LPL	lipoprotein lipase	NM_000237	-3.86
PCDH8	protocadherin 8	NM_002590	-3.85
MATK	megakaryocyte-associated tyrosine kinase	NM_002378	-3.84
CPLX2	complexin 2	NM_001008220	-3.84
JPH3	junctophilin 3	NM_020655	-3.82

References

1. Hodges A, Strand AD, Aragaki AK, Kuhn A, Sengstag T, Hughes G, Elliston LA, Hartog C, Goldstein DR, Thu D, Hollingsworth ZR, Collin F, Synek B, Holmans PA, Young AB, Wexler NS, Delorenzi M, Kooperberg C, Augood SJ, Faull RL, Olson JM, Jones L, Luthi-Carter R (2006) Regional and cellular gene expression changes in human Huntington's disease brain. Human molecular genetics 15 (6):965-977. doi:10.1093/hmg/ddl013

2. Fang H, Harris SC, Su Z, Chen M, Qian F, Shi L, Perkins R, Tong W (2009) ArrayTrack: an FDA and public genomic tool. Methods in molecular biology 563:379-398. doi:10.1007/978-1-60761-175-2_20

3. Pepper SD, Saunders EK, Edwards LE, Wilson CL, Miller CJ (2007) The utility of MAS5 expression summary and detection call algorithms. BMC bioinformatics 8:273. doi:10.1186/1471-2105-8-273

4. Shippy R, Fulmer-Smentek S, Jensen RV, Jones WD, Wolber PK, Johnson CD, Pine PS, Boysen C, Guo X, Chudin E, Sun YA, Willey JC, Thierry-Mieg J, Thierry-Mieg D, Setterquist RA, Wilson M, Lucas AB, Novoradovskaya N, Papallo A, Turpaz Y, Baker SC, Warrington JA, Shi L, Herman D (2006) Using RNA sample titrations to assess microarray platform performance and normalization techniques. Nature biotechnology 24 (9):1123-1131. doi:10.1038/nbt1241

5. Lamb J, Crawford ED, Peck D, Modell JW, Blat IC, Wrobel MJ, Lerner J, Brunet JP, Subramanian A, Ross KN, Reich M, Hieronymus H, Wei G, Armstrong SA, Haggarty SJ, Clemons PA, Wei R, Carr SA, Lander ES, Golub TR (2006) The Connectivity Map: using gene-expression signatures to connect small molecules, genes, and disease. Science 313 (5795):1929-1935. doi:10.1126/science.1132939

6. Smalley JL, Gant TW, Zhang SD (2010) Application of connectivity mapping in predictive toxicology based on gene-expression similarity. Toxicology 268 (3):143-146. doi:10.1016/j.tox.2009.09.014

7. Zhang SD, Gant TW (2008) A simple and robust method for connecting small-molecule drugs using gene-expression signatures. BMC bioinformatics 9:258. doi:10.1186/1471-2105-9-258

8. Mason RP, Casu M, Butler N, Breda C, Campesan S, Clapp J, Green EW, Dhulkhed D, Kyriacou CP, Giorgini F (2013) Glutathione peroxidase activity is neuroprotective in models of Huntington's disease. Nat Genet. doi:10.1038/ng.2732
